# Complete mitochondrial genome of *Liorhyssus hyalinus* (Hemiptera: Rhopalidae)

**DOI:** 10.1080/23802359.2022.2107456

**Published:** 2022-08-08

**Authors:** Qin Chen, Xiaoke Tian, Yongqin Li, Qianquan Chen

**Affiliations:** School of Life Sciences, Guizhou Normal University, Gui’an, China

**Keywords:** Mitogenome, hyaline grass bug, scentless plant bug

## Abstract

The mitogenome of *Liorhyssus hyalinus* (Fabricius, 1794), decoded using next-generation sequencing, is the first report of *Liorhyssus*. The mitogenomic size was 16,355 bp with 41.99% A, 33.44% T, 14.53% C, and 10.05% G (OM328158). The phylogenetic tree, constructed with the amino acid sequences of 13 protein-coding genes, showed that *L. hyalinus* clustered together with other species in Rhopalidae, which supported the monophyly of each family in Pentatomomorpha.

*Liorhyssus hyalinus* (Fabricius, 1794), with 20 synonymic names, is distributed across all continents except the coldest parts in the north and south (Hradil et al. 2007). It is found at elevations from 360 m below sea level to 3660 m, and causes serious damage to many low-growing crop plants, especially those in the family Asteraceae, by feeding primarily on the reproductive parts of plants, including buds, flowers, seeds, and fruits. Owing to their strong plasticity in size and coloration, identifying them based on their morphological characteristics is difficult (Hradil et al. 2007). The mitogenome of *L. hyalinus* was decoded to overcome this challenge.

Specimens of *L. hyalinus* were collected from the campus of Guizhou Normal University (26°23′11″N, 106°37′45″E) in July 2021. The specimens were deposited at the Museum of Guizhou Normal University (https://sjxy.gznu.edu.cn/info/1771/4547.htm, Qianquan Chen, qqchen@gznu.edu.cn) under voucher number GZNU-cqq-132. Total DNA was isolated from the muscle tissue of an adult specimen (Chen et al. 2020). The mitogenome was sequenced using the Illumina Hiseq X Ten System at Sangon Biotechnology Company (Shanghai, China). The mitogenome was assembled using SOAPdenovo2 (version 2.04) (Luo et al. 2012). Protein-coding genes (PCGs) were identified by BLAST comparison with the *Chorosoma macilentum* mitogenome (MN412594) (Zhao et al. 2021). The transfer RNAs (tRNAs) were identified with MITOS2 (Bernt et al. 2013), and ribosomal RNAs and non-coding control regions were determined by the boundary of tRNAs. A phylogenetic tree was constructed with the amino acid sequences of 13 PCGs belonging to the infraorder Pentatomomorpha, using the MrBayes method with partition models in PhyloSuite (Zhang et al. 2020). *Himacerus nodipes* (JF927832; Hemiptera: Nabidae) was selected as an outgroup representative.

The mitogenomic size of *L. hyalinus* (OM328158) was 16,355 bp. *Liorhyssus hyalinus* shared a gene distribution pattern similar to that of *C. macilentum* (MN412594). A total of 33 bp intergenic spacers were distributed across eight locations. The shortest intergenic spacer was 1 bp, whereas the longest was 19 bp. A total of 27 bp overlaps were distributed across six locations. The shortest overlap was 1 bp and the longest overlap was 8 bp. The non-coding control region was 1781 bp in length. The initiation codon for most PCGs was ATN, except for *cox1* (TTG). Seven genes used either T or TA as incomplete stop codons.

The phylogenetic tree showed that *L. hyalinus* clustered together with other species in Rhopalidae and grouped sibling species with *Stictopleurus subviridis*. The tree supported the monophyly of each family of Pentatomomorpha. The mitogenomic information on *L. hyalinus* can shed light on its identification and geographic origin (Figure 1).

**Figure 1.**
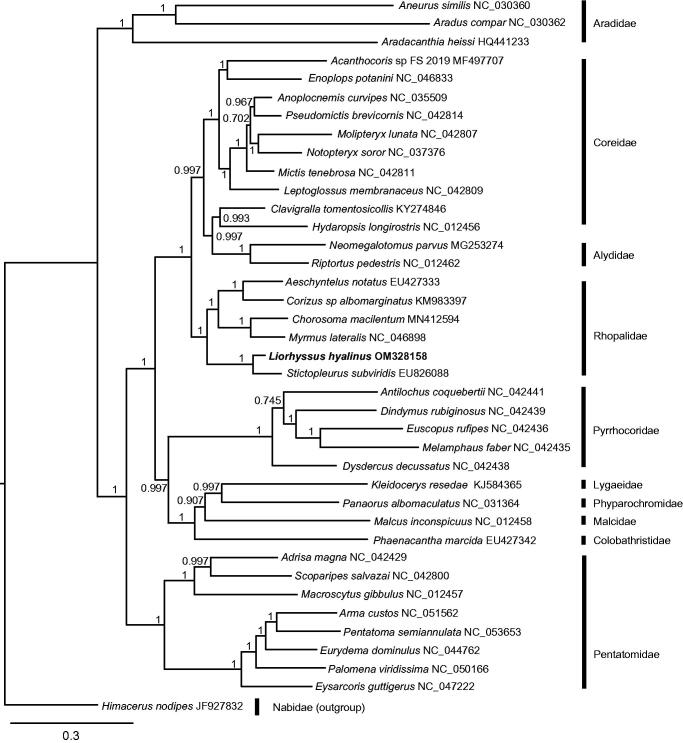
Bayesian phylogenetic tree of Pentatomomorpha species. The posterior probabilities are labeled at each node. The GenBank accession numbers of sequences are listed after the species name.

## Data Availability

The genome sequence data that support the findings of this study are openly available in GenBank (https://www.ncbi.nlm.nih.gov/) under accession no. OM328158. The associated BioProject, BioSample, and SRA numbers are PRJNA799448, SAMN25166436, and SRR17709601, respectively.
